# Pregnancy Requires Major Changes in the Quality of the Diet for Nutritional Adequacy: Simulations in the French and the United States Populations

**DOI:** 10.1371/journal.pone.0149858

**Published:** 2016-03-09

**Authors:** Clélia M. Bianchi, François Mariotti, Eric O. Verger, Jean-François Huneau

**Affiliations:** 1 UMR Physiologie de la Nutrition et du Comportement Alimentaire, AgroParisTech, INRA, Université Paris-Saclay, 75005, Paris, France; 2 INSERM, UMR_S U1166, Nutriomics team, F-75013, Paris, France; 3 Institute of Cardiometabolism and Nutrition, ICAN, Assistance Publique des Hôpitaux de Paris, Pitié-Salpêtrière hospital, Nutrition department, F-75013, Paris, France; University of São Paulo, BRAZIL

## Abstract

**Background:**

Maternal nutrition is critical to the health of both mother and offspring, but there is a paucity of data on the nutritional adequacy of diets during pregnancy.

**Objective:**

Our objective was to identify to what extent pregnancy reduces the nutritional adequacy of the expecting mother’s diet and if this nutritional gap can be resolved by simple quantitative or qualitative changes in the diet.

**Materials and Methods:**

We evaluated the observed overall nutritional adequacy of diets of French and American women of childbearing age participating in ENNS (n = 344) and NHANES (n = 563) using the probabilistic approach of the PANDiet system, resulting in a 100-point score. Then, we simulated the changes in the PANDiet scores of women of childbearing age who would remain on their diet during pregnancy. Finally, by either increasing the quantity of consumed foods or using eleven snacks recommended during pregnancy, we simulated the effect of a 150-kcal increase in the energy intake of French women.

**Results:**

Observed PANDiet scores were equal to 59.3 ± 7.0 and 58.8 ± 9.3 points respectively in France and in the US. Simulation of pregnancy for women of childbearing age led to a decrease in nutritional adequacy for key nutrients during pregnancy and resulted in reducing PANDiet scores by 3.3 ± 0.1 and 3.7 ± 0.1 points in France and in the US. Simulated 150-kcal increases in energy intake proved to be only partially effective in filling the gap both when the quantity of food consumed was increased and when recommended snacks were used.

**Conclusions:**

The decrease in nutritional adequacy induced by pregnancy cannot be addressed by simply following generic dietary guidelines.

## Introduction

During pregnancy, nutritional requirements increase to support both fetal growth and development and maternal metabolism and tissue accretion [1]. Poor maternal nutrition, as characterized by low intake or status in single nutrients during pregnancy, can result in adverse outcomes of pregnancy and birth [2–4]. The influence of the nutritional status may start even before pregnancy since preconceptional dietary patterns are associated with perinatal outcomes [5, 6]. On the other hand, several studies have reported that the intake of total energy, macronutrients [7] and a large set of vitamins and minerals [8, 9] are often inadequate in pregnant women in developed countries. Public health policies have been addressing maternal nutrition for several years. Indeed, dietary interventions, such as dietary counselling, are known to affect final critical outcomes for perinatal health [10]. The effect may however vary. For instance, in low-income pregnant women, recommendations on nutritional requirements or dietary advice to achieve national recommendations improve birth weight, whereas counselling to modify fat intake or meet energy requirements have the opposite effect [10]. Hence, there is critical need to analyze the impact of pregnancy on the nutritional gap between all nutrient intakes and requirements, to identify maternal nutrient intakes that have to be improved to optimize both neonatal and infant outcomes [10] and finally to evaluate the dietary strategy that can address this nutritional gap.

Overall diet quality of pregnant women and women of childbearing age can be evaluated using diet quality indexes that have been adapted to these specific populations [11–14]. However, because these indexes are based on food rather than nutrients, they do not relate specifically to the satisfaction of specific vitamin and mineral requirements during pregnancy.

To the best of our knowledge, nutritional adequacy estimates (based on nutrients) have never before been used to characterize the overall diet quality of pregnant women and the nutritional gap which exists during pregnancy. The Probability of Adequate Nutrient intake based Diet quality index (PANDiet) aims at measuring the overall diet quality through the probability of having adequate nutrient intakes for the largest possible set of nutrients [15]. It is versatile and adaptable to changes in specific nutritional requirements, such as those confronted during pregnancy.

The main objective of the present study was to evaluate the impact of pregnancy on the overall nutritional adequacy of womens’ diets. Using adapted PANDiet scoring, we evaluated the overall nutritional adequacy of the diet of French and US women of childbearing age and pregnant women participating in the French Nutrition and Health Survey (Etude Nationale Nutrition Santé—ENNS, 2006–2007) and in the National Health Administration and Nutrition Examination Survey (NHANES, 2009–2010). Then, we estimated the nutritional gap induced by pregnancy by simulating what the overall nutritional adequacy of the diet of women of childbearing age during the first trimester of pregnancy would be. Lastly, we evaluated the relevance of generic dietary guidelines for pregnancy by simulating their impact on the nutritional adequacy of the diet of French women of childbearing age.

## Materials and Methods

### Population study

Data used in this study came from ENNS (2006–2007) and NHANES (2009–2010).

The design and methodology of ENNS have already been described in detail elsewhere [16]. The ENNS survey was carried out according to the Declaration of Helsinki guidelines. All participants gave written informed consent and the survey protocol was approved by the Ethical Committee (Hôpital Cochin, Paris, no. 2264) and the French Data Protection Authority (Commission Nationale de l’Informatique et des Libertés, CNIL, authorization no.905481). The use of the anonymized data from the ENNS survey in this study has been granted to the authors by the main investigator of the ENNS survey (Unité de Surveillance en Epidémiologie Nutritionnelle, USEN). Briefly, the ENNS survey was a multistage stratified descriptive cross-sectional survey undertaken in 2006 and 2007. Non-institutionalized 18-74-year-olds living in mainland France were randomly selected. Dietary data were collected using three 24-hour recalls (including a weekend day) randomly selected within a 2-week period. Dietary recalls were conducted over the telephone by trained dieticians. Nutritional values for energy and nutrients were taken from a previously published nutrient database [17], updated to include recently marketed foods and data regarding the content in linoleic acid (LA), alpha-linolenic acid (ALA), docosahexaenoic acid (DHA), eicosapentaenoic acid (EPA), acid pantothenic, iodine and selenium. This update was conducted by extracting information from the French food composition table CIQUAL [18] and from the United States Department of Agriculture’s (USDA) National Nutrient Database for Standard Reference, Release 27 [19].

The design and methodology of NHANES have also been described in detail elsewhere [20]. All participants gave written informed consent and the survey protocol was approved by the National Center for Health Statistics Ethics Review Board. Anonymized data from the NHANES survey are publicly released and are freely available to download [21]. Briefly, the NHANES survey was a multistage stratified descriptive cross-sectional survey undertaken on a randomly selected sample of civilian non-institutionalized 20-80-year-olds living in the US. Subjects completed two 24-hour recalls (week or weekend days), the first of which was collected in-person by trained dieticians and the second over the telephone between three and ten days later. Nutritional values for energy and nutrients came from the USDA Food and Nutrient Database for Dietary Studies 5.0 (FNDDS 5.0) [22].

Subjects (i) who did not complete all food recalls (three for ENNS and two for NHANES), (ii) who had missing information about variables required to calculate the nutritional adequacy index (namely sex, age, bodyweight, menstruation, pregnancy) and (iii) who were identified as under-reporters based on the method proposed by Black *et al*. [23] were excluded from both surveys. In the studied samples, we selected only women who were pregnant (n = 22 in ENNS and n = 39 in NHANES) or of childbearing age, defined as premenopausal and under 45 years of age (n = 344 in ENNS and n = 563 in NHANES).

### Initial PANDiet

The PANDiet aims at measuring the overall diet quality of an individual by combining the probabilities of having an adequate intake in nutrients. Adequate intake was defined as the level likely to satisfy a nutrient requirement and unlikely to be excessive, according to dietary reference values, which differ with age, sex and physiological condition. The construction and design of the PANDiet have already been described in detail elsewhere [15]. Briefly, the PANDiet is a 100-point score that results from the average of two sub-scores, the Adequacy sub-score (AS) and the Moderation sub-score (MS). The higher the PANDiet is, the better the diet quality is. Each sub-score is composed of probabilities of adequacy for nutrients (21 for the AS and 6 for the MS), with a further 12 potential penalties for exceeding tolerable upper intake limits which are added to the MS. In its first version, 24 nutrients were considered: protein, total carbohydrate, dietary fibre, total fat, polyunsaturated fatty acids, saturated fatty acids, cholesterol, vitamin A, thiamin, riboflavin, niacin, vitamin B6, folate, vitamin B12, vitamin C, vitamin D and vitamin E, calcium, iron, magnesium, phosphorus, potassium, sodium and zinc [15]. The content and construct validities of the PANDiet had been previously evaluated for both the French and the US implementations. In brief, as far as the content validity is concerned, the correlations between the PANDiet score and the PANDiet items and the relationship between the PANDiet score and energy intake were investigated. As far as the construct validity is concerned, the PANDiet score was shown to be higher in individuals being non-smokers, consuming a lower-energy-dense diet and consuming more fruits and vegetables and less meat and processed meat [15].

### Implementation of the PANDiet for the French population

A probability of adequacy for the intake of free sugars was previously added to the MS in the implementation of the PANDiet for the French adults [24]. To further adapt the PANDiet for the present work, we added three nutrients: pantothenic acid, iodine and selenium, which resulted in the addition of three probabilities of adequacy to the AS and two potential penalties for iodine and selenium to the MS. Furthermore, the single probability of adequacy for PUFAs was replaced by four probabilities of adequacy for three fatty acids (LA, ALA and DHA) and for the sum of two fatty acids: EPA + DHA. In this study, the updated PANDiet score for the French sample was composed of 27 probabilities of adequacy (protein, total carbohydrate, dietary fibre, total fat, LA, ALA, DHA, EPA+DHA, vitamin A, thiamin, riboflavin, niacin, pantothenic acid, vitamin B6, folate, vitamin B12, vitamin C, vitamin D, vitamin E, calcium, iodine, iron, magnesium, phosphorus, potassium, selenium and zinc) in the AS and of seven probabilities of adequacy (protein, total carbohydrate, free sugars, total fat, saturated fatty acids, cholesterol and sodium) plus 14 potential penalties (retinol, niacin, vitamin B6, folate, vitamin C, vitamin D, vitamin E, calcium, iodine, iron, magnesium, phosphorus, selenium and zinc) in the MS.

Dietary reference values used to calculate the PANDiet for the French sample were those emitted by the French Agency for Food, Environmental and Occupational Health (ANSES), (S1 Table) [25, 26], except for iron for which the probability of adequacy was determined using values published by the Institute of Medicine [27, 28]. For some nutrients, different dietary reference values were proposed by the ANSES depending on the trimester of pregnancy. The validity of content and construct of the updated PANDiet score was assessed on French adults participating in ENNS (n = 1330) using the same method as for the initial scoring and this resulted in the same findings (data not shown).

### Implementation of the PANDiet for the US population

For the US implementation of the PANDiet, the probability of adequacy for selenium intake was added to the AS. The probability of adequacy for PUFAs intake in the AS was replaced by four probabilities of adequacy for LA, ALA, DHA and EPA+DHA intakes. A potential penalty corresponding to tolerable upper intake limit for selenium defined by the Institute of Medicine was also included in the MS. In this study, the updated PANDiet score for the US population was composed of 25 probabilities of adequacy in the AS (protein, total carbohydrate, total fat, LA, ALA, DHA, EPA+DHA, dietary fibre, vitamin A, thiamin, riboflavin, niacin, vitamin B6, folate, vitamin B12, vitamin C, vitamin D and vitamin E, calcium, iron, magnesium, phosphorus, potassium, selenium and zinc) and five probabilities of adequacy (total carbohydrate, total fat, saturated fatty acids, cholesterol and sodium) plus 12 potential penalties (retinol, niacin, vitamin B6, folate, vitamin C, vitamin D and vitamin E, calcium, iron, phosphorus, selenium and zinc) in the MS.

Dietary reference intakes used to calculate the PANDiet for the US population were those given by the Institute of Medicine (S2 Table) [27, 28], except for DHA and EPA+DHA. No dietary reference intakes were defined by the Institute of Medicine for DHA and EPA+DHA but given their association with pregnancy outcomes [29], dietary reference values for DHA and EPA+DHA were taken from the American Academy of Nutrition and Dietetics [30], which were indeed similar to the French dietary reference values [26]. Dietary reference intakes proposed by the IoM for pregnancy were the same whatever the trimester of pregnancy, except for protein.

### Diet recommendations for pregnant women

Energy requirements during pregnancy have been under discussion for a long time and recommendations differ according to countries and agencies [31–35]. In France, the guidelines consider an increase in energy intake for the first trimester of pregnancy with an approximate reference value at 150 kcal. In line with these guidelines, four snacks have been proposed to be added to the usual diet of pregnant women [36]. Agencies from some other countries have also proposed snacks for pregnancy. Therefore, seven healthy snacks for pregnancy proposed by British, Swiss and Quebecker public health agencies were secondly used in this analysis [37–39]. Food items composing these snacks are shown in Table 1. Because the energy density differed between the eleven snacks, we adjusted their weights so that they provide the same 150-kcal energy content. This adjustment was needed to compare the effect of the snacks for the same amount of energy.

**Table 1:** Composition, weight and 150-kcal adjusted weight of healthy snacks recommended during pregnancy.

Snack description	Snack content		150 kcal adjusted snack weight (g)	References^1^
	Composition	Weight (g)		
*Milk and soft bun*	A soft bun	35^2^	163	[36]
	A glass of half-skimmed milk	200^3^		
*Banana and yogurt*	A banana	150^2^	227	[36]
	A plain yogurt	125^2^		
*Bread with nuts*^4^	A little whole bread	50	44	[36]
	Walnuts	15		
*Cereal bar and yogurt*	A cereal bar	22.5^2^	143	[36]
	A plain yogurt	125^2^		
*Walnuts and yogurt*	A handful of walnuts	30^2^	91	[39]
	A plain yogurt	125^2^		
*Fruit and yogurt*	A fruit (average food^5^)	150^2^	286	[37, 38]
	A plain yogurt	125^2^		
*Bread and cheese*	A slice of whole bread	30^6^	47	[37]
	A slice of Emmental	30^6^		
*Toasts and egg*	Two toasts	16^2^	74	[39]
	A hard-boiled egg	55^2^		
*Vegetable sticks and hummus*	Carrot sticks	100^7^	153	[38]
	Hummus	30^7^		
*Bread with baked beans in tomato sauce*	A slice of whole bread	30^5^	103	[38]
	Baked beans	50^7^		
	Tomato sauce	10^7^		
*Pita bread filled with salad and tuna*	Half of a pita bread	35^2^	96	[38]
	Salad	15^7^		
	Mashed tuna	40^7^		

^1^ References refer to guidelines or documents for pregnant women edited by public health agencies in France [36], in the United Kingdom [38], in Switzerland [37], and in Quebec [39].

^2^ Weights of a soft bun, a plain yogurt, a banana, a cereal bar, a fruit, a handful of nuts, two toasts, a hard-boiled egg, and half of a pita bread were determined as an average of most commonly marketed foods in France (as supplied by the manufacturers or retailers).

^3^ Weight of the half-skimmed milk portion was determined as the equivalent of the content of one standard glass.

^4^ Weights of walnuts and bread composing the bread with nuts were determined as an average of recipes of little bread with nuts found in France.

^5^ Average food as presented in the French food composition table is a “theoretical” food, composition of which is estimated by a combination, weighted by levels of consumption, of several precise food items.

^6^ Weights of a slice of whole bread, a slice of Emmental correspond to servings “C” in the French guidebook for estimating food quantities [40].

^7^ Weights of carrots sticks and hummus in vegetable and hummus, of baked beans and tomato sauce in bread with baked beans in tomato sauce and of salad and tuna in pita bread filled with salad and mashed tuna were defined using common recipes.

### Calculating the PANDiet for women of childbearing age and pregnant women

As a first approach to describe the situation, observed PANDiet scores were calculated for women of childbearing age and pregnant women using the nutritional requirements respectively for premenopausal women and for women in the third trimester of pregnancy. As no information were available about the trimester of pregnancy in ENNS, nutritional requirements for the third trimester of pregnancy were used to calculate observed PANDiet scores of pregnant women to avoid a potential overestimation of nutritional adequacy for some women. The PANDiet is designed to focus on nutrient intakes from food alone. Even if national guidelines recommend the use of dietary supplements to meet requirements for some nutrients (folate, vitamin D, iron or iodine) during pregnancy, nutrient intakes from dietary supplements were not included in the PANDiet calculation, like in other studies using diet quality indexes for pregnancy [11–13].

### Simulating the PANDiet for women of childbearing age if they become pregnant—estimating the nutritional gap

Then, simulated-pregnancy PANDiet scores and associated sub-scores for women of childbearing age were also calculated using nutritional requirements for the first trimester of pregnancy. The difference between observed PANDiet and simulated-pregnancy PANDiet represents the decrease in nutritional adequacy induced by the change in physiological status of each individual, when keeping the same diet. In these simulations, we used nutritional requirements for the first trimester of pregnancy and did not extrapolate to the third trimester in order to focus on the transition period between the non-pregnant status and the first trimester of pregnancy to estimate the nutritional gap.

### Simulating the effects of French generic dietary guidelines on women of childbearing age—evaluating the theoretical efficiency for addressing the nutritional gap

Based on the simulated-pregnancy PANDiet scores, we simulated the effect of different changes in the diet, both quantitatively and qualitatively, with two types of simulations. The first one was a simple proportional increase in food weights consumed by each individual that resulted in a 150-kcal increase in energy intake. The second one consisted of the addition of one of the eleven snacks for pregnancy. However, at a practical level, in order not to increase their energy intake, pregnant women could add these snacks to their diet but consume less of the other foods composing their usual diet. To explore this possibility, a supplemental approach was implemented: one of the eleven snacks was used as a substitution for 150 kcal of the observed diets of women of childbearing age. US nutritional guidelines developed in the *MyPlate* initiative proposed a *Daily Food Plan for Moms* [41] but no healthy snacks to eat during pregnancy were identified. This approach is also consistent with the absence of guidelines regarding an increase in energy intake during pregnancy. Therefore, for the sake of consistency, this approach was not conducted in the US population.

### Statistical analyses

To describe the distribution of PANDiet scores, elemental statistics (mean, standard deviation, standard error of the mean and quartiles) were used. PANDiet scores and probabilities of adequacy are presented as mean ± SD. Differences between PANDiet scores are presented as mean ± SEM. Effects of physiological status (pregnancy / childbearing age) on PANDiet scores, AS, MS, energy intake and probabilities of adequacy were assessed by using Student’s t-tests after a Box-Cox transformation when residuals were not normally distributed. As pregnant women sample sizes were small, power calculations were performed for the comparisons between the scores of the pregnant women as compared to the scores of the women of childbearing age. Differences between observed and simulated-pregnancy PANDiet scores according to observed PANDiet quartiles were assessed by ANOVA. Multiple comparisons were then conducted using Bonferroni correction. Mixed models were used to analyze whether the PANDiet scores, AS, MS and probabilities of adequacy for nutrient intakes were different according to the nature of the simulation (pregnancy with/without increase in food consumption or snack addition) after a Box-Cox transformation when residuals were not normally distributed. All analyses were performed using SAS version 9.1.3 (SAS Institute Inc., Cary, NC). *P*<0.05 was considered significant.

## Results

### Preliminary observations: evaluation of the overall diet quality of women of childbearing age and pregnant women

Before simulating the impact of pregnancy on the diet of women of childbearing age, we ran a preliminary analysis to compare the observed overall diet quality of pregnant women (31.5 ± 5.2 y for the French sample and 29.6 ± 7.4 y for the US sample) and women of childbearing age (35.1 ± 6.9 y and 32.1 ± 7.4 y). Energy intakes, PANDiet scores, AS and MS (mean ± SD) for women of childbearing age and pregnant women in the French and the US population samples are shown in Table 2. In the French sample, the PANDiet scores and the AS were lower in pregnant women than in women of childbearing age, while the MS were not significantly different between the two groups. No differences were found between energy intakes of women of childbearing age and pregnant women.

**Table 2:** PANDiet scores, Adequacy sub-scores and Moderation sub-scores (mean ± SD) of the diets of French and US women.

	French population sample (ENNS^1^)		US population sample (NHANES^2^)	
	Women of childbearing age (n = 344)	Pregnant women (n = 22)	Women of childbearing age (n = 563)	Pregnant women (n = 39)
Energy intake^3^ (kcal/d)	1878 ± 402	1934 ± 444	2016 ± 499	2315* ± 661
PANDiet score	59.3 ± 7.0	54.5* ± 7.5	58.8 ± 9.3	59.9 ± 8.9
Adequacy sub-score	61.1 ± 11.9	54.7* ± 13.1	62.3 ± 11.2	63.5 ± 11.1
Moderation sub-score	57.4 ± 11.9	54.2 ± 13.7	55.3 ± 17.4	56.3 ± 17.5

^1^ ENNS, Etude Nationale Nutrition Santé.

^2^ NHANES, National Health Administration and Nutrition Examination Survey.

^3^ Excluding alcohol.

*P<0.05 as compared to women of childbearing age in the same country (Student’s t-test).

In the US sample, PANDiet scores, AS and MS were the same for women of childbearing age and pregnant women. However, the energy intake in the group of pregnant women was higher than in the group of women of childbearing age.

Probabilities of adequacy for nutrients composing the PANDiet for French and US pregnant women and women of childbearing age are available in S3 Table.

### Simulating the PANDiet for women of childbearing age if they become pregnant—estimating the nutritional gap

**Fig 1:**
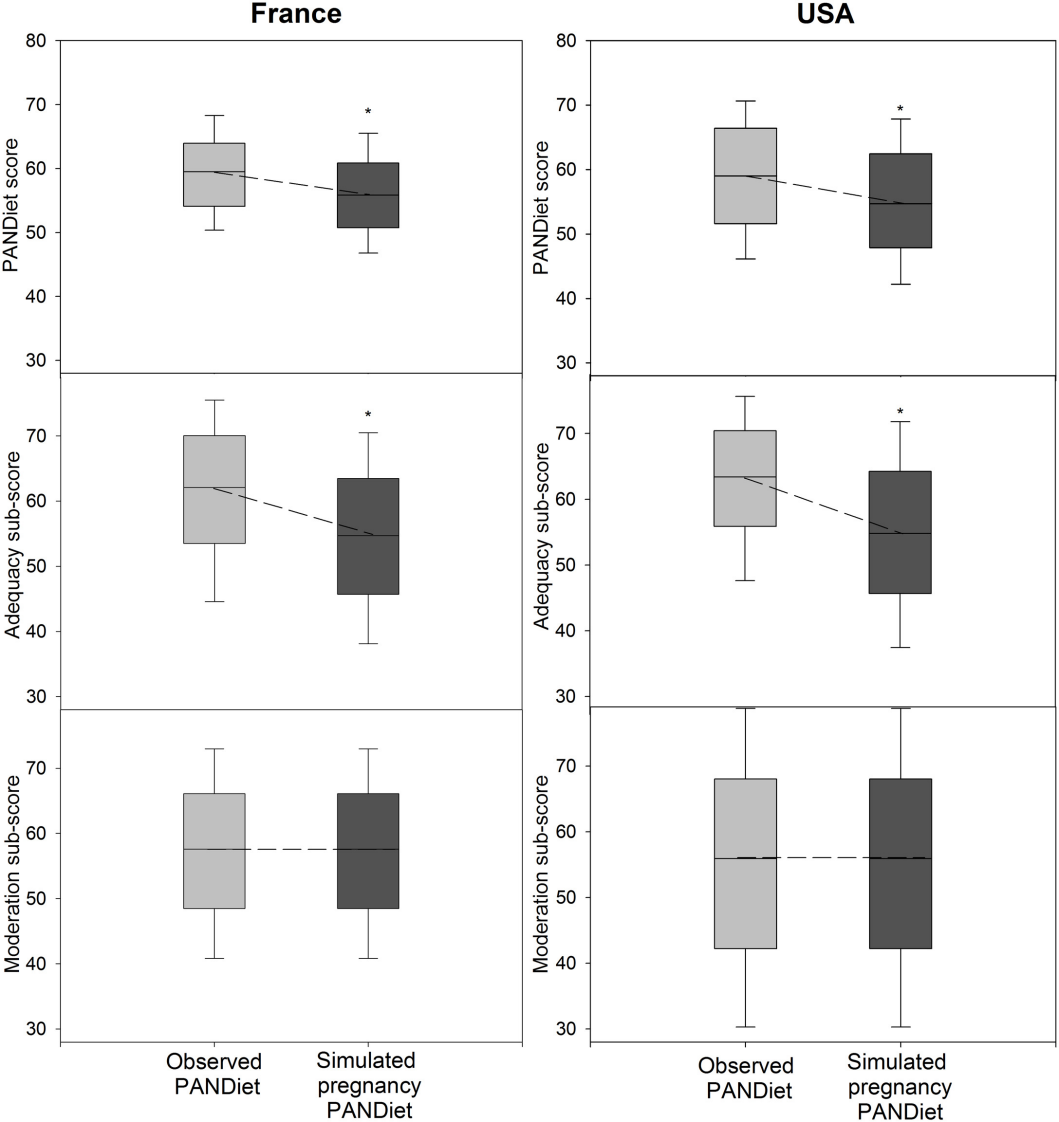
Distribution of observed PANDiet scores and simulated-pregnancy PANDiet scores, Adequacy sub-scores and Moderation sub-scores for French women of childbearing age from the ENNS survey (left panel) and US women of childbearing age from the NHANES survey 2009–2010 (right panel). Each bar represents n = 344 for the French sample and n = 563 for the US sample. The light grey bars represent the observed PANDiet score and sub-scores. The dark grey bars represent the simulated-pregnancy PANDiet score and sub-scores. The middle line in the box plots shows the median, the bottom and top of the box are the 25th and 75th percentiles and the ends of the whiskers represent the 5th and 95th percentiles. Differences between observed and simulated-pregnancy PANDiet scores, Adequacy sub-scores and Moderation sub-scores were assessed with a mixed model: * P<0.05

In women of childbearing age, simulating the pregnancy-induced changes in nutritional requirements led to a decrease in nutritional adequacy of the observed diet in both population samples (59.3 ± 7.0 for observed PANDiet scores vs 55.9 ± 7.3 for simulated-pregnancy PANDiet scores in the French sample, and 58.8 ± 9.3 vs 55.1 ± 9.8 in the US sample, Fig 1). Because reference dietary intakes for nutrients composing the MS did not differ in pregnancy, the MS remained unchanged in each population sample. In contrast, AS decreased markedly in both samples (Fig 1).

Table 3 shows variations between observed and simulated-pregnancy PANDiet scores according to the observed PANDiet score quartiles. A significant effect of observed PANDiet score quartiles on the variations between observed and simulated-pregnancy PANDiet scores was seen in the French sample and in the US sample.

**Table 3:** Differences between observed and simulated-pregnancy PANDiet scores (mean ± SEM) by observed PANDiet score quartiles among French and US women of childbearing age.

Observed PANDiet score quartile	Difference between observed and simulated-pregnancy PANDiet scores	
	French women of childbearing age^1^ (n = 344, ENNS^3^)	US women of childbearing age^2^ (n = 563, NHANES^4^)
Q1	-3.48^a^ ± 0.09	-3.98^c^ ± 0.14
Q2	-3.56^a^ ± 0.11	-3.87^c^^,^^d^ ± 0.14
Q3	-3.32^a^^,^^b^ ± 0.13	-3.59^c^^,^^d^ ± 0.17
Q4	-2.92^b^ ± 0.13	-3.38^d^ ± 0.17

^1^ Difference between observed and simulated-pregnancy PANDiet scores varied across observed PANDiet score quartiles in French women of childbearing age (ANOVA, *P* = 0.0006).

^a,b^ *P*<0.05: Multiple comparisons were conducted under Bonferroni correction.

^2^ Difference between observed and simulated-pregnancy PANDiet scores varied across observed PANDiet score quartiles in US women of childbearing age (ANOVA, *P* = 0.03).

^c,d^ *P*<0.05: Multiple comparisons were conducted under Bonferroni correction.

^3^ ENNS, French Nutrition and Health Survey (Etude Nationale Nutrition Santé)

^4^ NHANES, National Health Administration and Nutrition Examination Survey

**Fig 2:**
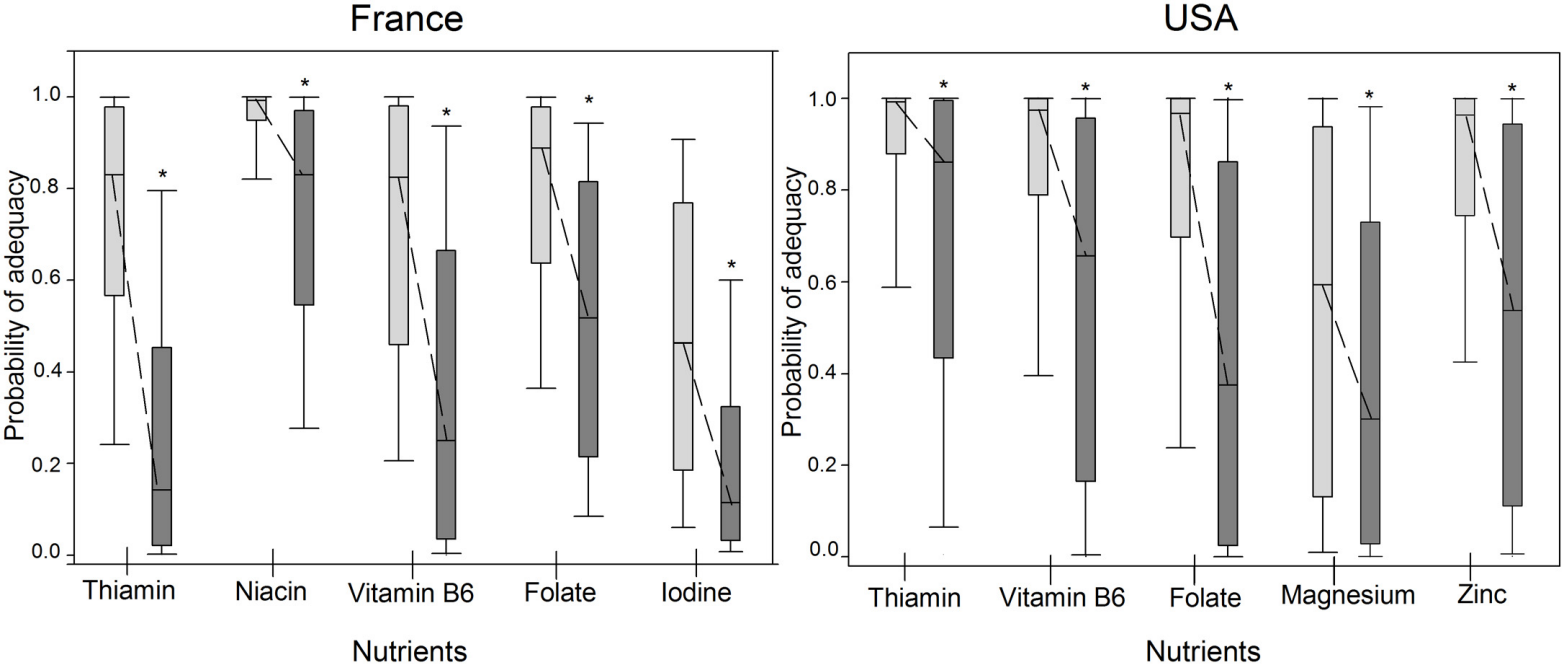
Probabilities of adequacy composing PANDiet scores for the five vitamins and minerals for which the probability of adequacy was lowered the most when simulating the changes in the nutritional requirements in the first trimester of pregnancy in the French sample (left panel) and in the US sample (right panel). Each bar represents n = 344 for the French sample and n = 563 for the US sample. The light grey bars represent probabilities of adequacy for nutrient intakes composing the observed PANDiet score. The dark grey bars represent probabilities of adequacy for nutrient intakes composing the simulated-pregnancy PANDiet score. The middle line in the box plots shows the median, the bottom and top of the box are the 25th and 75th percentiles and the ends of the whiskers represent the 5th and 95th percentiles. Differences between observed and simulated-pregnancy probabilities of adequacy for nutrients were assessed with a mixed model after a Box-Cox transformation: * P<0.05.

In simulations of the effect of pregnancy on nutritional adequacy, the decrease in the AS was explained by decreases in the probabilities of adequacy for some nutrients. Such was the case for 8 out of 34 nutrients (thiamin, riboflavin, niacin, vitamin B6, folate, vitamin D, iodine and zinc) for the French sample and 12 out of 30 (LA, ALA, vitamin A, thiamin, riboflavin, niacin, vitamin B6, folate, vitamin B12, vitamin C, magnesium and zinc) for the US sample (S4 Table). Fig 2 presents the five vitamins and minerals for which the probability of adequacy was lowered the most in the French sample (thiamin, niacin, vitamin B6, folate and iodine) and in the US sample (thiamin, vitamin B6, folate, magnesium and zinc).

### Simulating the effects of French generic dietary guidelines on women of childbearing age—evaluating the theoretical efficiency for addressing the nutritional gap

A 150-kcal increase in the intake of the diets of French women of childbearing age that would become pregnant was not effective in improving their diet quality (Table 4). This increase in energy intake increased the AS (+4.5 ± 0.9 points, P<0.01) and lowered the MS (-2.7 ± 0.9 points, P<0.01). The addition of any of the four 150-kcal adjusted snacks recommended by the French PNNS always led to an improvement of the simulated-pregnancy PANDiet score, even if the magnitude of the improvement differed according to the snack composition (Table 4). The addition of one of the seven 150-kcal adjusted snacks recommended by other national agencies led to more varied results depending on the snack. Although the addition of five of these snacks resulted in similar improvements, adding the ‘bread and cheese’ or the ‘egg and toasts’ snacks failed to increase the simulated-pregnancy PANDiet score. The best increase in the simulated-pregnancy PANDiet score (+5.15 ± 0.1 points) was obtained with the ‘banana and yogurt’ 150-kcal adjusted snack (Table 4). Finally, only five of the eleven generic dietary guidelines solved the nutritional gap induced by pregnancy in more than half of the population.

**Table 4:** Simulated-pregnancy PANDiet scores after simulations of a 150-kcal addition to the diet of French women of childbearing age from ENNS^1^ (n = 344).

Simulation of 150-kcal addition to women of childbearing age diets	Observed PANDiet score^2^	Simulated-pregnancy PANDiet score^2^	Differences in PANDiet score as compared to the simulated-pregnancy score^3^^,^^4^	Percentage of women whose nutritional gap was solved by the snack addition^5^
*Proportional increase in consumed food weight*	59.3 ± 7.0	55.9 ± 7.3	+0.94 ± 0.05	0.9%
***150-kcal adjusted snacks***				
*Milk and soft bun*			+1.54* ± 0.07	9.0%
*Banana and yogurt*			+5.15* ± 0.12	86.6%
*Bread with nuts*			+2.98* ± 0.07	37.8%
*Cereal bar and yogurt*			+3.22* ± 0.08	45.4%
*Walnuts and yogurt*			+3.34* ± 0.12	51.5%
*Fruit and yogurt*	59.3 ± 7.0	55.9 ± 7.3	+4.37* ± 0.10	73.0%
*Bread and cheese*			+0.22 ± 0.07	0.9%
*Toasts and egg*			-0.45 ± 0.12	2.3%
*Vegetable sticks and hummus*			+1.96* ± 0.10	20.1%
*Bread with baked beans in tomato sauce*			+3.34* ± 0.10	52.0%
*Pita bread filled with salad and tuna*			+3.91* ± 0.13	63.1%

^1^ ENNS, French Nutrition and Health Survey (Etude Nationale Nutrition Santé)

^2^ All values are mean ± SD.

^3^All values are mean ± SEM.

^4^ Differences between simulated-pregnancy PANDiet scores after simulation of 150-kcal additions to women of childbearing age diet were assessed with a mixed model:

*P<0.05, difference as compared to simulated-pregnancy PANDiet score before 150-kcal addition.

^5^ Nutritional gap was considered as solved when the simulated-pregnancy PANDiet score with the 150-kcal adjusted snack addition was superior or equal to the observed PANDiet score.

Depending on the snack content, simulations of snack additions had different effects on the probabilities of adequacy for different nutrients. By focusing on nutrients considered as key during pregnancy in French nutritional guidelines: folate, vitamin D, calcium, iodine and iron (36) plus DHA, we noted that three snack additions (‘cereal bar and yogurt’, ‘toasts and egg’ and ‘pita bread filled with salad and tuna’) and the proportional increase in consumed food weight increased probabilities of adequacy for four key nutrient intakes during pregnancy. However, the proportional increase in consumed food weight improved only slightly the overall nutritional adequacy and the ‘toasts and egg’ addition did not improve it. The eight other snack addition resulted in increasing probabilities of adequacy for two or three key nutrients during pregnancy. Probabilities of adequacy for those key nutrients during pregnancy obtained after each simulation are presented in S5 Table. When, rather than simulating addition, we simulated a substitution of the 150-kcal adjusted snacks for 150 kcal of the observed diet, we found lower effects on PANDiet scores. However, in seven out of eleven snacks, this substitution yielded an increase in the simulated-pregnancy PANDiet score (S6 Table).

## Discussion

This study provides the first estimate of the global nutritional gap as induced by pregnancy by using simulations from the diets of women of childbearing age. This study also offers clear evidence that resolving this nutritional gap requires above all major changes in the quality of the diet and not only an increase in energy intake. The nutritional gap associated to the onset of pregnancy is not fully addressed by the snacks currently recommended for pregnant women by public health agencies in developed countries.

Because there are mostly based on food alone, diet quality indexes do not make it possible to identify the nutritional gap induced by pregnancy. For instance, the American Healthy Eating Index for Pregnancy (AHEI-P) has reported similar scores between pregnant women and women of childbearing age. However, iron and folate requirements were met by a larger number of women of childbearing age than of pregnant women [11]. Hence, adequate assessment of diet quality in pregnancy requires also addressing the adequacy of nutrient intakes. Indeed, the mainstream in adapting diet quality indexes for pregnancy is to add items in the calculation of scores to describe the adherence to recommendations concerning pregnancy for important vitamins and minerals. Using this approach, the Mediterranean Diet Score for Pregnancy [13] and the Diet Quality Index [42] were adapted by adding three items evaluating the adherence of pregnant women to specific requirements for calcium, folate and iron intakes. However, as agreed by Laraia *et al*., the increase in nutritional requirements during pregnancy cannot be reduced to three nutrients [42]. For instance, folate requirements have been included in previously cited diet quality indexes because it is known to be strongly and positively associated with a decrease in the risk of neural tube defects. However, the risk of neural tube defects has also been associated with a diet quality scoring that takes into account more nutrients in addition to folate (calcium, iron, vitamin B6 and vitamin A, and percentage of calories from fat and from sweets) [43]. In contrast to these composite diet quality indexes based on dietary recommendations for adults and slightly updated with nutrient recommendations for pregnant women, the PANDiet index is versatile and can be directly adapted to the physiological status of a population by changing the scoring parameters to account for the specific set of nutritional requirements. It also uses the most comprehensive set of recommendations, and therefore gives the most global picture of the nutritional adequacy of the diet. It is particularly well adapted for studying the nutritional gap arising from pregnancy by using firstly the set of nutritional requirements for premenopausal women and, secondly, the set of nutritional requirements for women in their first trimester of pregnancy.

We found no other study simulating the effect of pregnancy on diet quality and we thus consider our estimate of the nutritional gap as induced by pregnancy to be a novel finding. Others studies on large cohort of pregnant women have focused on the nutritional adequacy during pregnancy, however nutrients were considered individually [44, 45]. These studies did not intend to provide an estimate of the overall diet quality of pregnant women.

Another finding of our study is that this pregnancy-induced nutritional gap cannot be addressed by a simple increase in energy. For the first trimester of pregnancy, the French guidelines consider an increase in energy intake with an approximate reference value at 150 kcal, but when simulating this increase, nutritional adequacy of the diet for pregnancy is not improved. As already stated, nutritional requirements that markedly increase during pregnancy [27, 28, 35] are those related to vitamins and minerals. Hence, the underlying reason of this failure to improve the PANDiet score is that initial diets of women of childbearing age are not rich enough in vitamins and minerals and too rich in sodium and cholesterol. Therefore, the quantitative increase in energy intake does not make it possible to meet the extra requirements during pregnancy, whereas it leads to a deterioration of the MS. Public health agencies develop generic dietary guidelines for pregnancy that are supposed to solve or limit the decrease in nutritional adequacy of the diet. We tested the four snacks that have been recommended in France and a set of seven other snacks, to provide a wide view of their theoretical efficiency. Our simulations show that most of the snacks result in an increase in nutritional adequacy of the diet, with two failing to do so when we simulated an addition to the diet and three when we simulated a 150-kcal energy substitution of the observed diet. However, the theoretical efficiency of these snacks to address the pregnancy-induced nutritional gap varied considerably from snacks that finally resulted in restoring the pre-pregnancy nutritional adequacy to a snack that resulted in a decrease in overall nutritional adequacy. The overall theoretical efficiency of some of these snack recommendations is not negligible, but it is clearly limited as compared to the improvement that may be expected from optimization, in particular if based on the observed diet of each individual. For instance, in the general population, we have shown that a substitution of two food items in initial diets of individuals results in an improvement of PANDiet scores that is higher than those found in the present simulation [24]. Further research is needed to evaluate the theoretical and practical efficiency of the current recommendations and propose systematic methods to select or define general or specific dietary advice based on its theoretical efficiency, which would yet be acceptable and thus practically efficient.

### Limitations

In both surveys, sample sizes of pregnant women were rather small and we cannot be sure that they are representative of the general population. Although we did find significant differences between the overall diet quality of French pregnant women and women of childbearing age, the statistical power of the comparisons remained limited. Given the sample sizes, we were able to detect a difference in PANDiet score of 4.3 points both in France and in the US with a correct power (beta = 0.20, alpha = 0.05). This comparison between the observed populations was however a preliminary assessment only, which does not at all concern the main findings from the simulation on the populations, with large sample size. Women who become pregnant have a physiological increase in nutritional requirements but as their nutrition awareness also rises, they are more likely to change their diets for a healthier pattern [46]. These changes are poorly predictable and difficult to delineate on the basis of observational data comparing pregnant women and women of childbearing age. A cohort study only could identify the real pregnancy-induced changes in the nutritional adequacy of womens' diets. By performing simulations of changes in the diet and the physiological status, we could delineate the specific effect of these changes on the nutritional adequacy of the diet, based on a comprehensive insight into each woman’s diet and we could also simulate the effect of specific simple changes in their diet. Such simulations however of course remain theoretical.

## Conclusion

Pregnancy tends to markedly widen the nutritional gap. The decrease in the nutritional adequacy of observed diets of women of childbearing age cannot be solved by a simple increase in energy intake, as recommended in some countries. A relatively good nutritional adequacy in pregnant women can therefore not be simply attributed to a higher energy intake, but to qualitative changes in the diet. Recommendations of snack additions from public health agencies make a somewhat limited and an extremely variable contribution to tackling the nutritional gap during pregnancy. These results call for dedicated studies to define the most theoretically efficient dietary counselling during pregnancy on a methodologically sound basis, either as generic counselling for the whole population or as tailor-fitted advice at the individual level, to improve the nutritional adequacy of the diet which is jeopardized at this specific, critical stage of the life cycle.

## Supporting Information

S1 Table Items, reference values^1^ and variabilities used in the French implementation of the updated PANDiet for women of childbearing age and women during the first and the third trimester of pregnancy. ^1^References values were emitted by the French Agency for Food, Environmental and Occupational Health (25, 26). The Adequacy sub-score is composed by 27 items and the Moderation sub-score is composed by 7 items plus 14 potential penalty values. ALA, Alpha Linolenic Acid. Bw, bodyweight. DHA, docosahexaenoic acid. EPA, eicosapentaenoic acid EIEA, Energy Intake Excluding Alcohol. LA, Linoleic Acid. NES, Niacin Equivalents. RE, Retinol Equivalents.
(DOCX)

S2 Table Items, reference values^1^ and variabilities used in the US implementation of the updated PANDiet for women of childbearing age and women during the first and the third trimester of pregnancy. ^1^References values were emitted by the Food and Nutrition Board of the United States Department of Agriculture (27, 28), except for EPA and DHA whose reference values come from the American Academy of Nutrition and Dietetics (30).
The Adequacy sub-score is composed by 25 items and the Moderation sub-score is composed by 5 items plus 12 potential penalty values. ALA, Alpha Linolenic Acid. Bw, bodyweight. DHA, docosahexaenoic acid. EPA, eicosapentaenoic acid. EIEA, Energy Intake Excluding Alcohol. LA, Linoleic Acid. NES, Niacin Equivalents. RE, Retinol Equivalents.
(DOCX)

S3 Table PANDiet scores, associated sub-scores and probabilities of adequacy for nutrients corresponding to observed PANDiet by country (France (ENNS) / USA (NHANES)) and physiological status (Women of childbearing age/ Pregnant women). ^1,2^ Differences between PANDiet scores, AS, MS and probabilities of adequacy by physiological status in the same country were assessed by using Student’s t-tests. A Box Cox transformation was used for probabilities of adequacy for nutrients because residuals were not normally distributed: *P<0.05; **P<0.01. ALA, Alpha-linolenic Acid. AS, Adequacy sub-score. DHA, docosahexaenoic acid. ENNS, French Nutrition and Health Survey (Etude Nationale Nutrition Santé). EPA, eicosapentaenoic acid. LA, linoleic acid. NA, not available. NHANES, National Health Administration and Nutrition Examination Survey.
(DOCX)

S4 Table Observed and simulated-pregnancy PANDiet scores, AS, MS and associated probabilities of adequacy among French women of childbearing age (n = 344) from ENNS and US women of child-bearing age (n = 563) from NHANES. Differences between observed and simulated-pregnancy PANDiet scores, AS, MS and probabilities of adequacy in the same country were assessed with a mixed model. A Box Cox transformation was used for probabilities of adequacy for nutrients when residuals were not normally distributed: *P<0.05; **P<0.01. ALA, Alpha-linolenic Acid. AS, Adequacy sub-score. DHA, docosahexaenoic acid. ENNS, French Nutrition and Health Survey (Etude Nationale Nutrition Santé). EPA, eicosapentaenoic acid. LA, linoleic acid. NA, not available. NHANES, National Health Administration and Nutrition Examination Survey.
(DOCX)

S5 Table Probabilities of adequacy for key nutrients during pregnancy (mean ± SD): DHA, Folate, Vitamin D, Calcium, Iodine and Iron obtained after different simulations (simulated-pregnancy without any addition, with a proportional increase in consumed food weight of 150-kcal or with the addition of one of the eleven 150-kcal snacks recommended during pregnancy) in French women of childbearing age (ENNS^1^, n = 344). ^1^ ENNS, French Nutrition and Health Survey (Etude Nationale Nutrition Santé). As probabilities of adequacy were not normally distributed, they were transformed using Box-Cox transformations before being used in the mixed model. *P<0.05 difference as compared to simulated-pregnancy probability of adequacy before simulation of 150-kcal addition assessed with a mixed model.
(DOCX)

S6 Table Simulated-pregnancy PANDiet scores obtained after substitutions of 150-kcal of the observed diet with snacks among French women of childbearing age from ENNS^1^ (n = 344). ^1^ ENNS, French Nutrition and Health Survey (Etude Nationale Nutrition Santé). ^2^ All values are mean ± SD. ^3^All values are mean ± SEM. ^4^ Differences between simulated-pregnancy PANDiet scores after simulation of 150-kcal substitutions in women of childbearing age diets were assessed with a mixed model: *P<0.05 difference as compared to simulated-pregnancy PANDiet score before 150-kcal substitution. ^5^ Nutritional gap was considered as solved when the simulated-pregnancy PANDiet score with the 150 kcal substitution with a snack was superior or equal to the observed PANDiet score.
(DOCX)

S7 Table STROBE Checklist. (DOCX)
