# Supplementary material for: Pregnancy Requires Major Changes in the Quality of the Diet for Nutritional Adequacy: Simulations in the French and the United States Populations
Source: PLoS One. 2016 Mar 9;11(3):e0149858. doi: 10.1371/journal.pone.0149858 (PMC4784858; doi:10.1371/journal.pone.0149858)
Supplement: S1 Table — 1References values were emitted by the French Agency for Food, Environmental and Occupational Health (25, 26). The Adequacy sub-score is composed by 27 items and the Moderation sub-score is composed by 7 items plus 14 potential penalty values. ALA, Alpha Linolenic Acid. Bw, bodyweight. DHA, docosahexaenoic acid. EPA, eicosapentaenoic acid EIEA, Energy Intake Excluding Alcohol. LA, Linoleic Acid. NES, Niacin Equivalents. RE, Retinol Equivalents. (DOCX) [file pone.0149858.s001.docx]

**S1 Table. Items, reference values^1^ and variabilities used in the French implementation of the updated PANDiet for women of childbearing age and women during the first and the third trimester of pregnancy.**

|  | Women of childbearing age | Pregnant women  (1^st^ trimester) | Pregnant women  (3^rd^ trimester) | Variability |
| --- | --- | --- | --- | --- |
| **Adequacy sub-score** | | | | |
| Protein | 0.66 g/kg bw | 0.66 g/kg bw | 0.8 g/kg bw | 12.50 % |
| Total Fat | 30% EIEA | 30% EIEA | 30% EIEA | 0 % |
| LA (C18:2 n-6) | 3.08 % EIEA | 3.08% EIEA | 3.08% EIEA | 15 % |
| ALA (C18:3 n-3) | 0.77% EIEA | 0.77% EIEA | 0.77% EIEA | 15 % |
| DHA | 192 mg | 192 mg | 192 mg | 15 % |
| EPA + DHA | 385 mg | 385 mg | 385 mg | 15 % |
| Total carbohydrate | 45 % EIEA | 45 % EIEA | 45 % EIEA | 0 % |
| Dietary fibre | 25 g | 25 g | 25 g | 15 % |
| Vitamin A | 462 µg RE | 462 µg RE | 539 µg RE | 15% |
| Thiamin | 0.85 mg | 1.39 mg | 1.39 mg | 15% |
| Riboflavin | 1.15 mg | 1.23 mg | 1.23 mg | 15 % |
| Niacin | 8.5 mg NES | 12.3 mg NES | 12.3 mg NES | 15 % |
| Pantothenic acid | 3.85 mg | 3.85 mg | 3.85 mg | 15 % |
| Vitamin B6 | 1.25 mg | 1.67 mg | 1.67 mg | 10 % |
| Folate | 215 µg | 286 µg | 286 µg | 20 % |
| Vitamin B12 | 2.0 µg | 2.17 µg | 2.17 µg | 10 % |
| Vitamin C | 84.6 mg | 92.3 mg | 92.3 mg | 15 % |
| Vitamin D | 3.85 µg | 7.70 µg | 7.70 µg | 15 % |
| Vitamin E | 9.23 mg | 9.23 mg | 9.23 mg | 15 % |
| Calcium | 690 mg | 690 mg | 760 mg | 15 % |
| Iodine | 107 µg | 143 µg | 143 µg | 20 % |
| Iron | Table provided by the IoM [27] | | | |
| Magnesium | 5 mg/kg bw | 5 mg/kg bw | 5.56 mg/kg bw | 10 % |
| Phosphorus | 580 mg | 580 mg | 640 mg | 15 % |
| Potassium | 2385 mg | 2385 mg | 2385 mg | 15 % |
| Selenium | 38.5 µg | 38.5 µg | 46.2 µg | 15 % |
| Zinc | 5.39 mg | 6.41 mg | 8.46 mg | 15 % |
| **Moderation sub-score** | | | | |
| Protein | 2.2 g/kg bw | 2.2 g/kg bw | 2.2 g/kg bw | 0 % |
| Total carbohydrate | 60% EIEA | 60% EIEA | 60% EIEA | 0 % |
| Free sugars | 10% EIEA | 10% EIEA | 10% EIEA | 15 % |
| Total fat | 40% EIEA | 40% EIEA | 40% EIEA | 0 % |
| Saturated Fatty Acids | 12% EIEA | 12% EIEA | 12% EIEA | 15 % |
| Cholesterol | 300 mg | 300 mg | 300 mg | 15 % |
| Sodium | 2365 mg | 2365 mg | 2365 mg | 15 % |
| *Tolerable Upper Intakes Limits for potential penalties* | | | | |
| Retinol | 3000 µg | | | |
| Niacin | 900 mg | | | |
| Vitamin B6 | 25 mg | | | |
| Folate | 1000 µg | | | |
| Vitamin C | 500 mg | | | |
| Vitamin D | 50 µg | | | |
| Vitamin E | 300 mg | | | |
| Calcium | 2500 mg | | | |
| Iodine | 600 µg | | | |
| Iron | 28 mg | | | |
| Magnesium | 700 mg | | | |
| Phosphorus | 2500 mg | | | |
| Selenium | 300 µg | | | |
| Zinc | 25 mg | | | |

^1^References values were emitted by the French Agency for Food, Environmental and Occupational Health [25, 26].

The Adequacy sub-score is composed by 27 items and the Moderation sub-score is composed by 7 items plus 14 potential penalty values.

ALA, Alpha Linolenic Acid. Bw, bodyweight. DHA, docosahexaenoic acid. EPA, eicosapentaenoic acid. EIEA, Energy Intake Excluding Alcohol. LA, Linoleic Acid. NES, Niacin Equivalents. RE, Retinol Equivalents
