# Supplementary material for: Pregnancy Requires Major Changes in the Quality of the Diet for Nutritional Adequacy: Simulations in the French and the United States Populations
Source: PLoS One. 2016 Mar 9;11(3):e0149858. doi: 10.1371/journal.pone.0149858 (PMC4784858; doi:10.1371/journal.pone.0149858)
Supplement: S2 Table — 1References values were emitted by the Food and Nutrition Board of the United States Department of Agriculture (27, 28), except for EPA and DHA whose reference values come from the American Academy of Nutrition and Dietetics (30). The Adequacy sub-score is composed by 25 items and the Moderation sub-score is composed by 5 items plus 12 potential penalty values. ALA, Alpha Linolenic Acid. Bw, bodyweight. DHA, docosahexaenoic acid. EPA, eicosapentaenoic acid. EIEA, Energy Intake Excluding Alcohol. LA, Linoleic Acid. NES, Niacin Equivalents. RE, Retinol Equivalents. (DOCX) [file pone.0149858.s002.docx]

**S2 Table. Items, reference values^1^ and variabilities used in the US implementation of the updated PANDiet for women of childbearing age and women during the first and the third trimester of pregnancy.**

|  | Women of childbearing age | Pregnant women (1^st^ trimester) | Pregnant women  (3^rd^ trimester) | | Variability | |
| --- | --- | --- | --- | --- | --- | --- |
| **Adequacy sub-score** | | | | | | |
| Protein | 0.66 g/kg bw | 0.66 g/kg bw | 0.88 g/kg bw | | 12 % | |
| Total Fat | 20% EIEA | 20% EIEA | 20% EIEA | | 0 % | |
| LA (C18:2 n-6) | 12 g | 13 g | 13 g | | 10 % | |
| ALA (C18:3 n-3) | 1.1 g | 1.4 g | 1.4 g | | 10 % | |
| DHA | 192 mg | 192 mg | 192 mg | | 15 % | |
| EPA + DHA | 385 mg | 385 mg | 385 mg | | 15 % | |
| Total carbohydrate | 45 % EIEA | 45 % EIEA | 45 % EIEA | | 0 % | |
| Dietary fibre | 14 g/1000 kcal | 14 g/1000 kcal | 14 g/1000 kcal | | 10 % | |
| Vitamin A | 500 µg RE | 550 µg RE | 550 µg RE | | 20 % | |
| Thiamin | 0.9 mg | 1.2 mg | 1.2 mg | | 10 % | |
| Riboflavin | 0.9 mg | 1.2 mg | 1.2 mg | | 10 % | |
| Niacin | 11 mg NES | 14 mg NES | 14 mg NES | | 10 % | |
| Vitamin B6 | 1.1 mg | 1.6 mg | 1.6 mg | | 10 % | |
| Folate | 320 µg | 520 µg | 520 µg | | 20 % | |
| Vitamin B12 | 2.0 µg | 2.2 µg | 2.2 µg | | 10 % | |
| Vitamin C | 60 mg | 70 mg | 70 mg | | 10 % | |
| Vitamin D | 10 µg | 10 µg | 10 µg | | 25 % | |
| Vitamin E | 12 mg | 12 mg | 12 mg | | 12.5 % | |
| Calcium | 800 mg | 800 mg | 800 mg | | 12.5 % | |
| Iron | Table provided by the IoM [27] | | | | | |
| Magnesium | 255 mg (19-30y)  265 mg (31-44y) | 290 mg (19-30y)  300 mg (31-44y) | 290 mg (19-30y)  300 mg (31-44y) | | 10 % | |
| Phosphorus | 580 mg | 580 mg | 580 mg | | 10 % | |
| Potassium | 4700 mg | 4700 mg | 4700 mg | | 10 % | |
| Selenium | 45 µg | 49 µg | 49 µg | | 10 % | |
| Zinc | 6.8 mg | 9.5 mg | 9.5 mg | | 10 % | |
| **Moderation sub-score** | | | | | | |
| Total carbohydrate | 65% EIEA | 65% EIEA | 65% EIEA | | 0 % | |
| Total fat | 35% EIEA | 35% EIEA | 35% EIEA | | 0 % | |
| Saturated Fatty Acids | 10% EIEA | 10% EIEA | 10% EIEA | | 10 % | |
| Cholesterol | 300 mg | 300 mg | 300 mg | | 10 % | |
| Sodium | 2300 mg | 2300 mg | 2300 mg | | 15 % | |
| *Tolerable Upper Intakes Limits for potential penalties* | | | | | | |
| Retinol | 3000 µg | | | | | |
| Niacin | 900 mg | | | | | |
| Vitamin B6 | 100 mg | | | | | |
| Folate | 1000 µg | | | | | |
| Vitamin C | 2000 mg | | | | | |
| Vitamin D | 100 µg | | | | | |
| Vitamin E | 1000 mg | | | | | |
| Calcium | 2500 mg | | | | | |
| Iron | 45 mg | | | | | |
| Phosphorus | 4000 mg | 3500 mg | | 3500 mg | |  |
| Selenium | 400 µg | | | | | |
| Zinc | 40 mg | | | | | |

^1^References values were emitted by the Food and Nutrition Board of the United States Department of Agriculture [27, 28], except for EPA and DHA whose reference values come from the American Academy of Nutrition and Dietetics [30].

The Adequacy sub-score is composed by 25 items and the Moderation sub-score is composed by 5 items plus 12 potential penalty values.

ALA, Alpha Linolenic Acid. Bw, bodyweight. DHA, docosahexaenoic acid. EPA, eicosapentaenoic acid. EIEA, Energy Intake Excluding Alcohol. LA, Linoleic Acid. NES, Niacin Equivalents. RE, Retinol Equivalents.
