# Supplementary material for: Pregnancy Requires Major Changes in the Quality of the Diet for Nutritional Adequacy: Simulations in the French and the United States Populations
Source: PLoS One. 2016 Mar 9;11(3):e0149858. doi: 10.1371/journal.pone.0149858 (PMC4784858; doi:10.1371/journal.pone.0149858)
Supplement: S3 Table — 1,2 Differences between PANDiet scores, AS, MS and probabilities of adequacy by physiological status in the same country were assessed by using Student’s t-tests. A Box Cox transformation was used for probabilities of adequacy for nutrients because residuals were not normally distributed: *P<0.05; **P<0.01. ALA, Alpha-linolenic Acid. AS, Adequacy sub-score. DHA, docosahexaenoic acid. ENNS, French Nutrition and Health Survey (Etude Nationale Nutrition Santé). EPA, eicosapentaenoic acid. LA, linoleic acid. NA, not available. NHANES, National Health Administration and Nutrition Examination Survey. (DOCX) [file pone.0149858.s003.docx]

**S3 Table. PANDiet scores, associated sub-scores and probabilities of adequacy for nutrients corresponding to observed PANDiet by country (France (ENNS) / USA (NHANES)) and physiological status (Women of Childbearing age/ Pregnant women).**

| **Country** | **France^1,2^** | | | **USA^1,2^** | | |
| --- | --- | --- | --- | --- | --- | --- |
| Physiological status | Women of childbearing age  (n=344) | Pregnant women  (n=22) |  | Women of childbearing age  (n=563) | Pregnant women  (n=39) |  |
| **PANDiet** | **59.3 ± 7.0** | **54.5 ± 7.5** | ****** | **58.8 ± 9.3** | **59.9 ± 8.9** |  |
| **AS** | **61.1 ± 11.9** | **54.7 ± 13.1** | ***** | **62.3 ± 11.2** | **63.5 ± 11.1** |  |
| *Total Carbohydrate* | 0.38 ± 0.39 | 0.25 ± 0.31 |  | 0.79 ± 0.33 | 0.82 ± 0.34 |  |
| *Total Fat* | 0.92 ± 0.18 | 0.98 ± 0.04 |  | 0.97 ± 0.13 | 0.97 ± 0.08 |  |
| *LA* | 0.60 ± 0.34 | 0.60 ± 0.32 |  | 0.63 ± 0.37 | 0.67 ± 0.38 |  |
| *ALA* | 0.09 ± 0.20 | 0.06 ± 0.12 |  | 0.66 ± 0.36 | 0.58 ± 0.39 |  |
| *DHA* | 0.17 ± 0.29 | 0.22 ± 0.37 |  | 0.07 ± 0.21 | 0.07 ± 0.21 |  |
| *EPA + DHA* | 0.15 ± 0.27 | 0.23 ± 0.37 |  | 0.05 ± 0.17 | 0.05 ± 0.19 |  |
| *Protein* | 0.97 ± 0.08 | 0.95 ± 0.08 |  | 0.90 ± 0.20 | 0.71 ± 0.29 | ****** |
| *Dietary fibre* | 0.12 ± 0.20 | 0.11 ± 0.20 |  | 0.11 ± 0.24 | 0.14 ± 0.27 |  |
| *Vitamin A* | 0.78 ± 0.27 | 0.64 ± 0.36 | ***** | 0.54 ± 0.37 | 0.72 ± 0.30 | ****** |
| *Thiamin* | 0.73 ± 0.29 | 0.32 ± 0.32 | ****** | 0.88 ± 0.22 | 0.90 ± 0.15 |  |
| *Riboflavin* | 0.85 ± 0.23 | 0.89 ± 0.16 |  | 0.95 ± 0.13 | 0.95 ± 0.14 |  |
| *Niacin* | 0.94 ± 0.13 | 0.81 ± 0.24 | ****** | 0.94 ± 0.15 | 0.94 ± 0.12 |  |
| *Pantothenic acid* | 0.64 ± 0.32 | 0.71 ± 0.30 |  | NA | | |
| *Vitamin B6* | 0.71 ± 0.31 | 0.45 ± 0.35 | ****** | 0.84 ± 0.26 | 0.73 ± 0.34 | ***** |
| *Folate* | 0.78 ± 0.26 | 0.53 ± 0.35 | ****** | 0.80 ± 0.30 | 0.64 ± 0.39 | ****** |
| *Vitamin B12* | 0.90 ± 0.18 | 0.88 ± 0.21 |  | 0.86 ± 0.24 | 0.86 ± 0.26 |  |
| *Vitamin C* | 0.48 ± 0.38 | 0.49 ± 0.37 |  | 0.53 ± 0.41 | 0.64 ± 0.41 |  |
| *Vitamin D* | 0.10 ± 0.22 | 0.04 ± 0.12 | ****** | 0.13 ± 0.23 | 0.26 ± 0.32 | ***** |
| *Vitamin E* | 0.54 ± 0.34 | 0.53 ± 0.35 |  | 0.12 ± 0.25 | 0.13 ± 0.27 |  |
| *Calcium* | 0.75 ± 0.30 | 0.74 ± 0.30 |  | 0.60 ± 0.38 | 0.85 ± 0.25 | ****** |
| *Iodine* | 0.47 ± 0.31 | 0.29 ± 0.27 | ****** | NA | | |
| *Iron* | 0.78 ± 0.18 | 0.81 ± 0.16 |  | 0.86 ± 0.16 | 0.94 ± 0.07 | ****** |
| *Magnesium* | 0.38 ± 0.36 | 0.14 ± 0.22 | ****** | 0.54 ± 0.38 | 0.61 ± 0.40 |  |
| *Potassium* | 0.64 ± 0.31 | 0.73 ± 0.29 |  | 0.03 ± 0.10 | 0.05 ± 0.16 |  |
| *Phosphorus* | 0.98 ± 0.06 | 0.99 ± 0.04 |  | 0.98 ± 0.07 | 1.00 ± 0.01 |  |
| *Selenium* | 0.68 ± 0.31 | 0.60 ± 0.29 |  | 0.96 ± 0.13 | 0.98 ± 0.03 |  |
| *Zinc* | 0.95 ± 0.11 | 0.80 ± 0.30 | ****** | 0.83 ± 0.26 | 0.68 ± 0.33 | ****** |
| **MS** | **57.4 ± 11.9** | **54.2 ± 13.7** |  | **55.3 ± 17.4** | **56.3 ± 17.5** |  |
| *Total carbohydrate* | 0.99 ± 0.07 | 1.00 ± 0.00 |  | 0.93 ± 0.18 | 0.91 ± 0.20 |  |
| *Total fat* | 0.55 ± 0.39 | 0.53 ± 0.40 |  | 0.58 ± 0.41 | 0.65 ± 0.40 |  |
| *Saturated Fatty Acids* | 0.14 ± 0.21 | 0.09 ± 0.16 |  | 0.41 ± 0.37 | 0.50 ± 0.36 |  |
| *Cholesterol* | 0.45 ± 0.34 | 0.36 ± 0.33 |  | 0.68 ± 0.37 | 0.66 ± 0.39 |  |
| *Protein* | 0.97 ± 0.11 | 0.99 ± 0.03 |  | NA | | |
| *Free sugars* | 0.56 ± 0.38 | 0.63 ± 0.36 |  | NA | | |
| *Sodium* | 0.36 ± 0.31 | 0.23 ± 0.28 | ***** | 0.16 ± 0.24 | 0.11 ± 0.19 |  |
| *Penalty* | 0.02 ± 0.14 | 0.05 ± 0.21 |  | 0.02 ± 0.14 | 0.05 ± 0.22 |  |

^1,2^ Differences between PANDiet scores, AS, MS and probabilities of adequacy by physiological status in the same country were assessed with Student’s t-tests. A Box Cox transformation was used for probabilities of adequacy for nutrients because residuals were not normally distributed: **P*<0.05 ; ***P*<0.01.

ALA, Alpha-linolenic Acid. AS, Adequacy sub-score. DHA, docosahexaenoic acid. ENNS, French Nutrition and Health Survey (Etude Nationale Nutrition Santé). EPA, eicosapentaenoic acid. LA, linoleic acid. NA, not available. NHANES, National Health Administration and Nutrition Examination Survey.
