# Supplementary material for: Pregnancy Requires Major Changes in the Quality of the Diet for Nutritional Adequacy: Simulations in the French and the United States Populations
Source: PLoS One. 2016 Mar 9;11(3):e0149858. doi: 10.1371/journal.pone.0149858 (PMC4784858; doi:10.1371/journal.pone.0149858)
Supplement: S4 Table — Differences between observed and simulated-pregnancy PANDiet scores, AS, MS and probabilities of adequacy in the same country were assessed with a mixed model. A Box Cox transformation was used for probabilities of adequacy for nutrients when residuals were not normally distributed: *P<0.05; **P<0.01. ALA, Alpha-linolenic Acid. AS, Adequacy sub-score. DHA, docosahexaenoic acid. ENNS, French Nutrition and Health Survey (Etude Nationale Nutrition Santé). EPA, eicosapentaenoic acid. LA, linoleic acid. NA, not available. NHANES, National Health Administration and Nutrition Examination Survey. (DOCX) [file pone.0149858.s004.docx]

**S4 Table.** **Observed and simulated-pregnancy PANDiet scores, AS, MS and associated probabilities of adequacy among French women of child-bearing age (n=344) from ENNS and US women of child-bearing age (n=563) from NHANES.**

|  | French women of childbearing age (n=344) | | | US women of childbearing age  (n=563) | | |
| --- | --- | --- | --- | --- | --- | --- |
|  | Observed PANDiet | Simulated pregnancy PANDiet |  | Observed PANDiet | Simulated pregnancy PANDiet |  |
|  |  |  |  |  |  |  |
| **PANDiet score** | **59.3 ± 7.0** | **55.9 ± 7.3** | ***** | **58.8 ± 9.3** | **55.1 ± 9.8** | ***** |
| **AS** | **61.1 ± 11.9** | **54.5 ± 12.5** | ***** | **62.3 ± 11.2** | **54.9 ± 13.3** | ***** |
| *Total carbohydrate* | 0.38 ± 0.39 | 0.38 ± 0.39 |  | 0.79 ± 0.33 | 0.79 ± 0.33 |  |
| *Total fat* | 0.92 ± 0.18 | 0.92 ± 0.18 |  | 0.97 ± 0.13 | 0.97 ± 0.13 |  |
| *ALA* | 0.09 ± 0.20 | 0.09 ± 0.20 |  | 0.66 ± 0.36 | 0.48 ± 0.20 | ** |
| *LA* | 0.60 ± 0.34 | 0.60 ± 0.34 |  | 0.63 ± 0.37 | 0.58 ± 0.38 | ** |
| *DHA* | 0.17 ± 0.29 | 0.17 ± 0.29 |  | 0.07 ± 0.21 | 0.07 ± 0.21 |  |
| *EPA + DHA* | 0.15 ± 0.27 | 0.15 ± 0.27 |  | 0.05 ± 0.17 | 0.05 ± 0.17 |  |
| *Protein* | 0.97 ± 0.08 | 0.97 ± 0.08 |  | 0.90 ± 0.20 | 0.90 ± 0.20 |  |
| *Dietary Fibre* | 0.12 ± 0.20 | 0.12 ± 0.20 |  | 0.11 ± 0.24 | 0.11 ± 0.24 |  |
| *Vitamin A* | 0.78 ± 0.27 | 0.78 ± 0.27 |  | 0.54 ± 0.37 | 0.48 ± 0.38 | ** |
| *Thiamin* | 0.73 ± 0.29 | 0.27 ± 0.30 | ** | 0.88 ± 0.22 | 0.70 ± 0.35 | ** |
| *Riboflavin* | 0.85 ± 0.23 | 0.80 ± 0.26 | ** | 0.95 ± 0.13 | 0.83 ± 0.27 | ** |
| *Niacin* | 0.94 ± 0.13 | 0.73 ± 0.28 | ** | 0.94 ± 0.15 | 0.87 ± 0.24 | ** |
| *Pantothenic Acid* | 0.64 ± 0.32 | 0.64 ± 0.32 |  | NA | | |
| *Vitamin B6* | 0.71 ± 0.31 | 0.37 ± 0.35 | ** | 0.84 ± 0.26 | 0.57 ± 0.38 | ** |
| *Folate* | 0.78 ± 0.26 | 0.52 ± 0.32 | ** | 0.80 ± 0.30 | 0.44 ± 0.40 | ** |
| *Vitamin B12* | 0.90 ± 0.18 | 0.88 ± 0.20 |  | 0.86 ± 0.24 | 0.83 ± 0.28 | ** |
| *Vitamin C* | 0.48 ± 0.38 | 0.43 ± 0.38 |  | 0.53 ± 0.41 | 0.47 ± 0.41 | ** |
| *Vitamin D* | 0.10 ± 0.22 | 0.03 ± 0.12 | ** | 0.13 ± 0.23 | 0.13 ± 0.23 |  |
| *Vitamin E* | 0.54 ± 0.34 | 0.54 ± 0.34 |  | 0.12 ± 0.25 | 0.12 ± 0.25 |  |
| *Calcium* | 0.75 ± 0.30 | 0.75 ± 0.30 |  | 0.60 ± 0.38 | 0.60 ± 0.38 |  |
| *Iodine* | 0.47 ± 0.31 | 0.22 ± 0.24 | ** | NA | | |
| *Iron* | 0.78 ± 0.18 | 0.78 ± 0.18 |  | 0.86 ± 0.16 | 0.86 ± 0.16 |  |
| *Magnesium* | 0.38 ± 0.36 | 0.38 ± 0.36 |  | 0.54 ± 0.38 | 0.40 ± 0.37 | ** |
| *Phosphorus* | 0.98 ± 0.06 | 0.98 ± 0.06 |  | 0.98 ± 0.07 | 0.98 ± 0.07 |  |
| *Potassium* | 0.64 ± 0.31 | 0.64 ± 0.31 |  | 0.03 ± 0.10 | 0.03 ± 0.10 |  |
| *Selenium* | 0.68 ± 0.31 | 0.68 ± 0.31 |  | 0.96 ± 0.13 | 0.95 ± 0.15 |  |
| *Zinc* | 0.95 ± 0.11 | 0.89 ± 0.18 | ** | 0.83 ± 0.26 | 0.53 ± 0.39 | ** |
| **MS** | **57.4 ± 11.9** | **57.4 ± 11.9** |  | **55.3 ± 17.4** | **55.3 ± 17.4** |  |
| *Protein* | 0.97 ± 0.11 | 0.97 ± 0.11 |  | NA | | |
| *Total carbohydrate* | 0.99 ± 0.07 | 0.99 ± 0.07 |  | 0.93 ± 0.18 | 0.93 ± 0.18 |  |
| *Free sugars* | 0.56 ± 0.38 | 0.56 ± 0.38 |  | NA | | |
| *Total fat* | 0.55 ± 0.39 | 0.55 ± 0.39 |  | 0.58 ± 0.41 | 0.58 ± 0.41 |  |
| *Saturated fat* | 0.14 ± 0.21 | 0.14 ± 0.21 |  | 0.41 ± 0.37 | 0.41 ± 0.37 |  |
| *Cholesterol* | 0.45 ± 0.34 | 0.45 ± 0.34 |  | 0.68 ± 0.37 | 0.68 ± 0.37 |  |
| *Sodium* | 0.36 ± 0.31 | 0.36 ± 0.31 |  | 0.16 ± 0.24 | 0.16 ± 0.24 |  |
| *Penalty* | 0.02 ± 0.14 | 0.02 ± 0.14 |  | 0.02 ± 0.14 | 0.02 ± 0.14 |  |

Differences between observed and simulated-pregnancy PANDiet scores, AS, MS and probabilities of adequacy in the same country were assessed with a mixed model. A Box Cox transformation was used for probabilities of adequacy for nutrients when residuals were not normally distributed: **P*<0.05 ; ***P*<0.01.

ALA, Alpha-linolenic Acid. AS, Adequacy sub-score. DHA, docosahexaenoic acid. ENNS, French Nutrition and Health Survey (Etude Nationale Nutrition Santé). EPA, eicosapentaenoic acid. LA, linoleic acid. NA, not available. NHANES, National Health Administration and Nutrition Examination Survey.
