# Supplementary material for: Pregnancy Requires Major Changes in the Quality of the Diet for Nutritional Adequacy: Simulations in the French and the United States Populations
Source: PLoS One. 2016 Mar 9;11(3):e0149858. doi: 10.1371/journal.pone.0149858 (PMC4784858; doi:10.1371/journal.pone.0149858)
Supplement: S5 Table — 1 ENNS, French Nutrition and Health Survey (Etude Nationale Nutrition Santé). As probabilities of adequacy were not normally distributed, they were transformed using Box-Cox transformations before being used in the mixed model. *P<0.05 difference as compared to simulated-pregnancy probability of adequacy before simulation of 150-kcal addition assessed with a mixed model. (DOCX) [file pone.0149858.s005.docx]

**S5 Table. Probabilities of adequacy for key nutrients during pregnancy (mean ± SD): DHA, Folate, Vitamin D, Calcium, Iodine and Iron obtained after different simulations (simulated-pregnancy without any addition, with a proportional increase in consumed food weight of 150-kcal or with the addition of one of the eleven 150-kcal snacks recommended during pregnancy) in French women of childbearing age (ENNS^1^, n=344)**

|  | **DHA** | **Folate** | **Vitamin D** | **Calcium** | **Iodine** | **Iron** | |
| --- | --- | --- | --- | --- | --- | --- | --- |
| *Simulated-pregnancy without any addition* | 0.17 ± 0.29 | 0.52 ± 0.32 | 0.03 ± 0.12 | 0.75 ± 0.30 | 0.22 ± 0.24 | 0.78 ± 0.18 | |
| **Simulated-pregnancy with a 150-kcal addition** | | | | | | |  |
| *Proportional increase in consumed food weight* | 0.19 ± 0.30 | 0.60* ± 0.31 | 0.04 ± 0.13 | 0.81* ± 0.26 | 0.27* ± 0.26 | 0.83* ± 0.15 | |
| ***150-kcal snacks*** | | | | | | | |
| *Milk and soft bun* | 0.18 ± 0.29 | 0.60* ± 0.30 | 0.03 ± 0.12 | 0.90* ± 0.18 | 0.32* ± 0.27 | 0.81 ± 0.16 | |
| *Banana and yogurt* | 0.17 ± 0.29 | 0.62* ± 0.30 | 0.03 ± 0.12 | 0.92* ± 0.16 | 0.42* ± 0.28 | 0.81 ± 0.16 | |
| *Bread with nuts* | 0.17 ± 0.29 | 0.58* ± 0.31 | 0.03 ± 0.12 | 0.77 ± 0.28 | 0.22 ± 0.24 | 0.83* ± 0.14 | |
| *Cereal bar and yogurt* | 0.17 ± 0.29 | 0.73* ± 0.26 | 0.03 ± 0.12 | 0.92* ± 0.16 | 0.42* ± 0.28 | 0.83* ± 0.14 | |
| *Walnuts and yogurt* | 0.17 ± 0.29 | 0.61* ± 0.30 | 0.03 ± 0.12 | 0.87* ± 0.21 | 0.31* ± 0.27 | 0.81 ± 0.15 | |
| *Fruit and yogurt* | 0.17 ± 0.29 | 0.54 ± 0.31 | 0.03 ± 0.13 | 0.93* ± 0.15 | 0.42* ± 0.28 | 0.80 ± 0.16 | |
| *Bread and cheese* | 0.17 ± 0.29 | 0.54 ± 0.31 | 0.04* ± 0.14 | 0.95* ± 0.12 | 0.26 ± 0.26 | 0.81 ± 0.16 | |
| *Toasts and egg* | 0.23* ± 0.32 | 0.68* ± 0.28 | 0.03 ± 0.12 | 0.80 ± 0.27 | 0.37* ± 0.25 | 0.85* ± 0.13 | |
| *Vegetable sticks and hummus* | 0.17 ± 0.29 | 0.72* ± 0.26 | 0.03 ± 0.12 | 0.84* ± 0.24 | 0.23 ± 0.25 | 0.86* ± 0.11 | |
| *Bread with baked beans in tomato sauce* | 0.17 ± 0.29 | 0.73* ± 0.26 | 0.03 ± 0.12 | 0.80 ± 0.27 | 0.23 ± 0.25 | 0.89* ± 0.09 | |
| *Pita bread filled with salad and tuna* | 0.25* ± 0.33 | 0.69* ± 0.27 | 0.05* ± 0.15 | 0.79 ± 0.27 | 0.26 ± 0.25 | 0.87* ± 0.11 | |

^1^ ENNS, French Nutrition and Health Survey (Etude Nationale Nutrition Santé)

As probabilities of adequacy were not normally distributed, they were transformed using Box-Cox transformations before being used in the mixed model.

**P*<0.05 difference as compared to simulated-pregnancy probability of adequacy before simulation of 150-kcal addition assessed with a mixed model.
