# Supplementary material for: Pregnancy Requires Major Changes in the Quality of the Diet for Nutritional Adequacy: Simulations in the French and the United States Populations
Source: PLoS One. 2016 Mar 9;11(3):e0149858. doi: 10.1371/journal.pone.0149858 (PMC4784858; doi:10.1371/journal.pone.0149858)
Supplement: S6 Table — 1 ENNS, French Nutrition and Health Survey (Etude Nationale Nutrition Santé). 2 All values are mean ± SD. 3All values are mean ± SEM. 4 Differences between simulated-pregnancy PANDiet scores after simulation of 150-kcal substitutions in women of childbearing age diets were assessed with a mixed model: *P<0.05 difference as compared to simulated-pregnancy PANDiet score before 150-kcal substitution. 5 Nutritional gap was considered as solved when the simulated-pregnancy PANDiet score with the 150 kcal substitution with a snack was superior or equal to the observed PANDiet score. (DOCX) [file pone.0149858.s006.docx]

**S6 Table. Simulated-pregnancy PANDiet scores obtained after substitutions of 150-kcal of the observed diet with snacks among French women of childbearing age from ENNS^1^ (n=344)**

| **Simulation of substituting 150 kcal of women of childbearing age diets by snacks** | **Observed PANDiet score^2^** | **Simulated-pregnancy PANDiet score^2^** | **Differences in PANDiet score as compared to the simulated-pregnancy score^3,4^** | **Percentage of women whose nutritional gap was solved by 150-kcal substitution with a snack^5^** |
| --- | --- | --- | --- | --- |
| *Milk and soft bun* |  |  | +0.74 ± 0.05 | 2.3% |
| *Banana and yogurt* |  |  | +4.57* ± 0.10 | 76.2% |
| *Bread with nuts* |  |  | +2.11* ± 0.06 | 23.3% |
| *Cereal bar and yogurt* |  |  | +2.44* ± 0.06 | 24.7% |
| *Walnuts and yogurt* |  |  | +2.38* ± 0.12 | 32.9% |
| *Fruit and yogurt* | 59.3 ± 7.0 | 55.9 ± 7.3 | +3.72* ± 0.09 | 58.1% |
| *Bread and cheese* |  |  | -0.66 ± 0.06 | 0.3% |
| *Toasts and egg* |  |  | -1.65* ± 0.12 | 0.3% |
| *Vegetable sticks and hummus* |  |  | +0.98 ± 0.09 | 10.5% |
| *Bread with baked beans in tomato sauce* |  |  | +2.47* ± 0.09 | 33.7% |
| *Pita bread filled with salad and tuna* |  |  | +3.45* ± 0.10 | 48.0% |

^1^ ENNS, French Nutrition and Health Survey (Etude Nationale Nutrition Santé)

^2^ All values are mean ± SD.

^3^All values are mean ± SEM.

^4^ Differences between simulated-pregnancy PANDiet scores after simulation of 150-kcal substitutions in women of childbearing age diet were assessed with a mixed model:

**P*<0.05 difference as compared to simulated-pregnancy PANDiet score before 150-kcal substitution.

^5^ Nutritional gap was considered as solved when the simulated-pregnancy PANDiet score with the 150 kcal substitution with a snack was superior or equal to the observed PANDiet score.
