# Supplementary material for: Pregnancy Requires Major Changes in the Quality of the Diet for Nutritional Adequacy: Simulations in the French and the United States Populations
Source: PLoS One. 2016 Mar 9;11(3):e0149858. doi: 10.1371/journal.pone.0149858 (PMC4784858; doi:10.1371/journal.pone.0149858)
Supplement: S7 Table — (DOCX) [file pone.0149858.s007.docx]

**S7 Table. STROBE Checklist**

|  | **Item No** | **Recommendation** |
| --- | --- | --- |
| **Title and abstract** | 1 | (*a*) Indicate the study’s design with a commonly used term in the title or the abstract: **l.1,2 (title:** **p.1)** |
|  |  | (*b*) Provide in the abstract an informative and balanced summary of what was done and what was found: **l.25-41 (abstract: p.2,3)** |
| **Introduction** | | |
| Background/rationale | 2 | Explain the scientific background and rationale for the investigation being reported: **l.43-70 (p.4,5)** |
| Objectives | 3 | State specific objectives, including any prespecified hypotheses: **l.71-80 (p.5)** |
| **Methods** | | |
| Study design | 4 | Present key elements of study design early in the paper: **l.84 (p.5); l.114-120 (p.6, 7); l.225-265 (p.11-13)** |
| Setting | 5 | Describe the setting, locations, and relevant dates, including periods of recruitment, exposure, follow-up, and data collection: **l.85-120 (p.5-7)** |
| Participants | 6 | (*a*) *Cross-sectional study*—Give the eligibility criteria, and the sources and methods of selection of participants : **l.84 (p.5) – l.114-120 (p.6, 7)** |
|  |  | (*b*) *Cohort study*—For matched studies, give matching criteria and number of exposed and unexposed : **NA**  *Case-control study*—For matched studies, give matching criteria and the number of controls per case : **NA** |
| Variables | 7 | Clearly define all outcomes, exposures, predictors, potential confounders, and effect modifiers. Give diagnostic criteria, if applicable: **l.114-120 (p.6, 7); l.144-167 (p.8, 9); l.169-189 (p.9); l.191-202 (p.10)** |
| Data sources/ measurement | 8* | For each variable of interest, give sources of data and details of methods of assessment (measurement). Describe comparability of assessment methods if there is more than one group: : **l.84 (p.5);** **l.114-120 (p.6, 7); l.144-167 (p.8, 9); l.169-189 (p.9); l.225-265 (p.11-13)** |
| Bias | 9 | Describe any efforts to address potential sources of bias: **l.225-237 (p.11,12)** |
| Study size | 10 | Explain how the study size was arrived at**: l.114-120 (p.6,7)** |
| Quantitative variables | 11 | Explain how quantitative variables were handled in the analyses. If applicable, describe which groupings were chosen and why: **l.227-229 (p.12);** **l.241-243 (p.12); l.253-261 (p.13); l.268-273 (p. 13, 14); l.276-283 (p.14)** |
| Statistical methods | 12 | (*a*) Describe all statistical methods, including those used to control for confounding : **l.268-283 (p.13,14)** |
|  |  | (*b*) Describe any methods used to examine subgroups and interactions: **l.271-282 (p.13,14) ;** |
|  |  | (*c*) Explain how missing data were addressed: **NA** |
|  |  | (*d*) *Cohort study*—If applicable, explain how loss to follow-up was addressed: **NA**  *Case-control study*—If applicable, explain how matching of cases and controls was addressed: **NA**  *Cross-sectional study*—If applicable, describe analytical methods taking account of sampling strategy: **NA** |
|  |  | (*e*) Describe any sensitivity analyses: **NA** |

| **Results** | | |
| --- | --- | --- |
| Participants | 13* | (a) Report numbers of individuals at each stage of study—eg numbers potentially eligible, examined for eligibility, confirmed eligible, included in the study, completing follow-up, and analysed : **NA** |
|  |  | (b) Give reasons for non-participation at each stage: **NA** |
|  |  | (c) Consider use of a flow diagram: **NA** |
| Descriptive data | 14* | (a) Give characteristics of study participants (eg demographic, clinical, social) and information on exposures and potential confounders: **l.286-289 (p.14) ;** |
|  |  | (b) Indicate number of participants with missing data for each variable of interest: **NA** |
|  |  | (c) *Cohort study*—Summarise follow-up time (eg, average and total amount) : **NA** |
| Outcome data | 15* | *Cohort study*—Report numbers of outcome events or summary measures over time: **NA** |
|  |  | *Case-control study—*Report numbers in each exposure category, or summary measures of exposure**: NA** |
|  |  | *Cross-sectional study—*Report numbers of outcome events or summary measures: **l.291-293 (p.14); l.313-316 (p. 16); l.335-338 (p.16, 17); l.355-362 (p.17, 18); l.382-389 (p.19); l.411-425 (p.20,21);** |
| Main results | 16 | (*a*) Give unadjusted estimates and, if applicable, confounder-adjusted estimates and their precision (eg, 95% confidence interval). Make clear which confounders were adjusted for and why they were included: **l.291-309 (p.14, 15); l.313-316 (p.16); l.335-352 (p.16, 17); l.355-362 (p.17, 18); l.382-408 (p.19, 20); l.411-425 (p.20, 21);** |
|  |  | (*b*) Report category boundaries when continuous variables were categorized:**l.341-352 (p.17)** |
|  |  | (*c*) If relevant, consider translating estimates of relative risk into absolute risk for a meaningful time period: **NA** |
| Other analyses | 17 | Report other analyses done—eg analyses of subgroups and interactions, and sensitivity analyses: **l.421-425 (p. 20, 21)** |
| **Discussion** | | |
| Key results | 18 | Summarise key results with reference to study objectives: **l.428-433 (p.21)** |
| Limitations | 19 | Discuss limitations of the study, taking into account sources of potential bias or imprecision. Discuss both direction and magnitude of any potential bias: **l.495-512 (p.23, 24)** |
| Interpretation | 20 | Give a cautious overall interpretation of results considering objectives, limitations, multiplicity of analyses, results from similar studies, and other relevant evidence: **l.434-493 (p.21-23)** |
| Generalisability | 21 | Discuss the generalisability (external validity) of the study results: **l.451-460 (p. 22); l.476-479 (p.23); l.490-493 (p.23)** |
| **Other information** | | |
| Funding | 22 | Give the source of funding and the role of the funders for the present study and, if applicable, for the original study on which the present article is based: **NA** |
